# Education Research: Rewarding Our Educators: Design and Evaluation of a Program to Fund Teaching in Neurology

**DOI:** 10.1212/NE9.0000000000200182

**Published:** 2024-12-30

**Authors:** Susannah Cornes, LeeAnn Chang, Sally Collins, Megan Richie, Audrey Foster-Barber, S. Andrew Josephson, Ann Poncelet

**Affiliations:** 1 Department of Neurology, University of California, San Francisco;; 2 Allegheny Health Network, Pittsburgh, PA; and; 3 School of Medicine, University of California, San Francisco.

## Abstract

**Background and Objectives:**

Pressures on academic physician educators to generate clinical revenue or research grants may threaten faculty engagement as teachers. Neurology has historically prized its outstanding educators, but programs that provide financial support for teaching are lacking. We developed an opt-in, financial, teaching incentive program in an academic neurology department and evaluated its impact on faculty experience, motivation, and identity.

**Methods:**

We applied a change management framework and conducted a National and local needs assessment before designing an education value unit (EVU) system using time-based metrics and rates reflecting impact on clinical revenue. Activities essential for graduation of students and residents that reduced clinical revenue generation were included. Faculty self-reported teaching through an online platform and received an incentive payment at the end of each year. Following a 6-month pilot, faculty participated in a 3-year implementation phase followed by an evaluation through faculty survey and semistructured interviews.

**Results:**

In the first 3 years of the program, 42, 56, and 54 faculty enrolled, representing 28% of faculty by year 3. Faculty reported an average of 1,488 hours of teaching annually, drew from 13 divisions, and included all ranks (51% assistant, 29% associate, and 20% full). Fifty-five percent of participating faculty (N = 30) completed a survey to evaluate the incentive program. The majority agreed or somewhat agreed that the program had met its goals (80%–92%), reduced barriers to teaching (56%), and the department highly valued teaching (93%). Semistructured interviews with 11 participating faculty identified 5 themes regarding the impact of the program on faculty experience, including (1) supporting the choice of faculty to teach even when time is scarce, (2) making teaching visible to oneself and others, (3) directing faculty toward eligible teaching opportunities, (4) communicating the department's commitment to education, and (5) reinforcing educator identity and sense of belonging.

**Discussion:**

The development of a teaching incentive program at an academic neurology center is feasible with benefits extending beyond the incentive payment itself. Although EVU programs are not without limitations, faculty experienced the program as reflective of the department valuing its educators, which reinforced their educator identity and engagement in teaching.

## Introduction

Pressures on academic physician educators to generate clinical revenue or research grants may threaten the commitment of faculty to medical education with teaching perceived as an unfunded mandate. Although neurology as a discipline has historically prized its outstanding educators, few academic neurology programs fund direct teaching,^1^ and neurology is not well-represented in a review of programs that support teaching.^2^ A sustainable teaching plan should include an investment of tangible support for teaching and allow for an iterative, transparent, and mission-based approach to align funding with departmental values.

This approach, known as mission-based management of educational funding, was proposed by the Association of American Medical Colleges (AAMC) in 1998, but the early concept suffered from a lack of feasibility. Proposed metrics to measure faculty effort and contributions to the education mission were complex, including scholarship, administration, teaching time, effort, and expertise.^3^ Program implementation quickly highlighted challenges for early adopters, such as the misguided search for a perfect metric system, the inability to erase ambiguities regarding contributions, and a tendency to become overly complex.^4^ Since that time, various institutions have adapted education value unit (EVU) systems,^2,5,6^ including time or point metrics,^7-10^ but have often suffered from being unsustainable or unpopular.^11^ Relatively few have been well-received by the faculty they intend to support.^12,13^

It has been challenging to evaluate the success of programs that support physician educators. Outcomes have focused on scholarly work, impact on salary, and teaching quality,^2^ potentially functioning to evaluate rather than support faculty.^14^ A weakness of program evaluation to date is the lack of focus on whether the program is truly incentivizing teaching by addressing faculty experience and motivation, the latter rooted in the need for self-determination, relatedness, and competence.^15-17^ Programs that undermine these factors—reducing faculty sense of agency or their communities of practice—could backfire. In addition, although there are calls for monetary incentives to teach, as opposed to other forms of incentive, monetary payments could paradoxically reduce motivation by undermining the conception of teaching as an altruistic act.^18,19^

For teaching incentive programs to be successful, we propose that they must (1) shift financial resources to support the teaching mission, (2) be feasible to implement and maintain, and (3) demonstrate a positive impact on faculty experience, motivation, and identity as an outcome measure. We developed an opt-in teaching incentive program in our academic neurology department that focused on self-reported core teaching and minimized administrative burden. After a 3-year implementation phase, we evaluated faculty perceptions regarding the success of the program and its effect on their experience, motivation, and identity as educators using a survey and semistructured interviews focused on these themes.

## Methods

### Designing the Program

Applying the Kotter 8 Steps for Leading Change model, we communicated a sense of urgency to the department chair of the need to address a growing reluctance of neurology faculty to teach. This led to the formation of a working group (the Neurology Committee on Sustainable Teaching or NCOST) with broad stakeholder representation across clinical sites and disciplines. The NCOST chair position (A.P. and then S.C.) was supported through the University of California, San Francisco Academy of Medical Educators (Rowe Endowed Chair). To develop our EVU program, called the Neurology Teaching EVU and Community Hub (NTEACH), the committee conducted needs assessments including (1) a survey of the consortium of neurology clerkship directors, (2) a survey of department faculty, and (3) a comprehensive inventory of all departmental teaching to estimate the total size of the educational mission.

Neurology Clerkship Director survey responses included 11 schools and identified supporting the educational mission of neurology departments as a struggle at most schools. Since that time, a broader survey of Department Chairs yielding similar data has been published.^1^ The survey of our own departmental faculty regarding barriers to teaching and changes that would be incentivizing yielded 31 respondents (17% responder rate). Faculty identified several barriers related to time (87%), financial (58%), clinical (58%), and research (52%) demands. Faculty recommended 4 mechanisms to support teachers: financial incentives, community building, advancement, and infrastructure.

Following this needs assessment, the committee created 5 program goals to guide us in our program development and iteration: (1) to enable faculty interested in teaching to do so without undue financial disincentive; (2) to promote teaching quality; (3) to foster career advancement for educators; (4) to reinvigorate a culture of education within the department; and (5) to actively realign resources with educational priorities, incentivizing educators to participate in teaching activities essential for the graduation of our medical students and residents.

The committee completed a comprehensive inventory of teaching activities across clinical sites and phases of education, including preclerkship, clerkship, and graduate medical education that included estimates for the total hours of the entire teaching endeavor and impact on clinical revenue. Educational activities were divided into 4 categories: (1) direct teaching, (2) administrative roles (clerkship director), (3) education scholarship, and (4) other educational roles or activities (such as faculty development; Table 1). Core learners were medical students and residents. Direct teaching was further subdivided into 3 groups: (1a) essential core teaching (necessary to meet programmatic requirements for medical students and residents), (1b) nonessential core (medical student and resident teaching not essential to meet programmatic requirements), and (1c) teaching of other groups beyond medical students and residents (such as fellowship teaching).

**Table 1:** Departmental Teaching Inventory

Teaching codes	Definition	Example
1a	Essential core	Neuroscience course, clerkship teaching
1b	Nonessential core	Grand rounds, elective rotation clinics
1c	Nonessential noncore	ACGME fellow teaching
2	Administrative roles	Site director roles, mentoring
3	Scholarship	Peer-reviewed publications in education
4	Mission priority	Medical education faculty development

Abbreviation: ACGME = Accreditation Council for Graduate Medical Education.

An inventory of departmental teaching was completed and coded to identify the subset of activities for inclusion in the program. The program's initial scope included only essential core (1a) activities. Select mission priority activities (4) were added during the implementation (e.g., Medical Education Faculty Development and Professor's Rounds).

The group used a consensus-building approach to develop time metrics for faculty reporting for each activity (e.g., 1 hour of teaching time per half-day of clinic with a medical student; Table 2). We next identified which teaching activities were relative value unit (RVU) generating or neutral (such as being an inpatient service attending), RVU decreasing (such as teaching medical students in an outpatient clinic), or absent RVUs (such as giving a lecture or leading a small group). Recognizing that essential core (1a) activities that decreased RVUs or did not generate RVUs were particularly in need of incentives to be sustainable in the current academic and health care environment, the group chose to focus the scope of the program on this category of teaching and generated a rubric to calculate teaching EVUs from the standard time metric multiplied by rate metric (Table 2). The focus of the program time-metric and incentive design were chosen to enable feasibility, transparency, sustainability, and equity.

**Table 2:** Incentivized Teaching Program Rubric

Category	Activity	Hour/activity	EVU rate/hour
Preclerkship didactic	Physical examination skills facilitator	2	1
	Preclerkship small group	2	1
	Preclerkship apprenticeship	3	1
	Preclerkship lecturer	1	1
Medical student clinics	Student clinic shadowing	1	0.5
	Student clinic independent	2	0.5
	Longitudinal student clinic	2	0.5
	Subintern student clinic	1	0.5
Medical student and	Clerkship student feedback	0.5	0.25
Resident evaluations	Clerkship observed examination	1	0.25
	Resident milestones evaluation	1	0.25
	Resident other evaluation	0.5	0.25
	Resident observed examination	1	0.25
Didactics	Clerkship core didactics	1	1
	Resident core didactic	1	1
Other priorities	Faculty development	1	1

Abbreviations: EVU = education value unit; NTEACH = Neurology Teaching EVU and Community Hub; RVU = relative value unit.

Program rubric reflecting core teaching category and activity for inclusion in NTEACH. Teaching activities were assigned a standard time metric and a multiplier rate intended to account for relative RVU impact.

Using the teaching inventory and rubric, NCOST developed an estimate of cost to support essential core teaching of medical students and residents, communicated the program vision to the department faculty and leadership, and secured a major financial contribution from the department chair to support the program through a 3-year implementation phase. Each year, the chair dedicates funds from central departmental resources for the program. Rate-per-time is determined by the annual budget set by the chair and aims to align end-of-year incentive payments with clinical rates. The chair of the department was one of the initial committee members who conceptualized this program, has cited this as one of his most important accomplishments in his recent 5-year stewardship review, and is an author (S.A.J.) on this article.

### Program Implementation and Continuous Improvement

NCOST developed a mechanism for monthly, opt-in self-reporting by faculty entered through computer or mobile phone into a departmental Smartsheet and offered faculty development for enrollment. Faculty were invited to enroll in the NTEACH pilot in 2018 followed by launch in 2019 with a process for iterative refinement and feedback on the reporting process. We maintained a faculty portal to bring feedback and recommendations to NCOST and developed a process to guide the annual evaluation of core teaching for inclusion within the program that included consideration for whether the activity was defined as “1a” essential core (as above), the number of faculty and hours for the activity, and whether it was undersubscribed. We tracked the total estimated hours of teaching covered, total hours reported, number of faculty enrolled, and faculty demographics to inform decisions regarding annual program budget. We generated an annual report to the chair and brought recommendations for programmatic changes before a departmental leadership committee.

Iterative changes to align with mission priorities over time included adding faculty development activities that supported teaching quality, such as those focused on diversity, equity, and inclusion, and adding Professor's Rounds teaching. To support faculty advancement and program experience, we added annual thank you letters, mobile access, and a monthly reminder mechanism.

### Program Evaluation

At the end of our 3-year implementation phase, we conducted a mixed-methods evaluation of the program, including (1) a faculty survey regarding the success of NTEACH in meeting its program goals and (2) semistructured faculty interviews to explore the impact on faculty of engagement in a teaching incentive program, including the impact on educator identity, motivation, and engagement in teaching (eAppendix 1).

We developed and piloted an interview guide with 1 faculty and then revised the guide before the remaining 10 interviews (conducted by S.C. between March and June 2022). Interviews asked for faculty to reflect on their experience with NTEACH, any changes in their teaching engagement during their participation, their educator identity, and factors that motivated them to teach. Interviews were conducted by S.C., transcribed using a professional transcription service and reviewed by the interviewer (S.C.) for accuracy. The first 3 interviews were independently analyzed by 3 researchers (L.C., S.C., and S.C.C.) using open coding and reconciled to create an initial codebook. Interviews were then coded by S.C.C. and one other researcher (L.C. or S.C.). The 3 researchers met iteratively to reconcile the codes and modify the codebook as needed. Coded interviews were uploaded into Dedoose (version 8.0.35; SocioCultural Research Consultants, LLC, Los Angeles, CA) for qualitative analysis. We used Braun and Clarke's methodology to analyze coded interviews for themes inductively.^20^ Coded transcripts were each analyzed by S.C.C. and one other researcher (L.C., A.P. or A.F.B.), and the team met regularly to discuss and refine themes and determine saturation.

### Standard Protocol Approvals, Registrations, and Patient Consents

This study was granted exempt status by the University of California, San Francisco Institutional Review Board (IRB# 20-30789). Consent forms were emailed to the faculty listserv. After reviewing the consent, faculty could proceed to the study survey and interview scheduling process. Verbal consent was repeated at the start of each interview.

### Data Availability

Anonymized data not published in this article will be made available on request from any qualified investigator.

## Results

### Program Demographics

After completion of our 3-year implementation phase, demographics for participants in the program revealed faculty diversity across ranks, divisions, inpatient and outpatient setting, and undergraduate and graduate medical education focus (Table 3). The majority of participants were at the assistant professor rank (assistant 51%, associate 29%, and full 20%). There was broad inclusion across the department with 10/10 divisions and 3/3 clinical sites represented. Of teaching hours reported, nearly half were from outpatient clinics (42%), followed by inpatient (27%) and classroom-based (24%) teaching (eTable 1). Faculty participation stabilized over 3 years and represented approximately 25% of eligible faculty.

**Table 3:** Participant Characteristics During Pilot and 3-Year Implementation Phase

	Pilot	Year 1	Year 2	Year 3
Participants, (%) n	15 (29)	21 (42)	29 (56)	28 (54)
Total hours	406	1,297	1,701	1,468
Rank, (%) n				
Assistant	55 (16)	48 (20)	57 (32)	46 (25)
Associate	34 (10)	31 (13)	23 (13)	33 (18)
Full	10 (3)	21 (9)	18 (10)	20 (11)

Program demographic data demonstrated relatively stable total enrollment during implementation as a percentage of eligible faculty. Assistant professors consistently made up the largest subset of participants.

### General Program Evaluation

Thirty faculty completed the program evaluation survey (55% of participants; eAppendix 2). The majority of faculty agreed or somewhat agreed that the program had met its 5 goals (1) to enable everyone interested in teaching to do so (92%), (2) to promote teaching quality (84%), (3) to foster career advancement (80%), (4) to reinvigorate a culture of education (88%), and (5) to actively realign resources with educational priorities (92%). Most felt the program had reduced barriers to teaching (56%) but did not feel the program had improved individual teaching (76%). The great majority of faculty (93%) felt that the department highly valued teaching.

### Impact on Faculty Identity, Engagement, and Motivation

Thematic analysis of the 11 faculty semistructured interviews revealed 5 themes regarding the faculty experience as educators and impact of NTEACH on engagement, identity, and motivation: (1) Faculty acknowledged the challenge of competing demands and felt that the program supported their choice to teach. (2) Faculty noted that participation in the incentive program helped make their teaching visible to themselves and others. (3) The program helped direct faculty toward eligible teaching opportunities and was feasible for faculty to engage in. (4) Faculty viewed the program as communicating the department's commitment to the educational mission and felt that the department valued their work as teachers. (5) Overall, faculty experienced the existence of the teaching incentive program as reinforcing their identity as educators and sense of belonging.

#### Supported the Choice to Teach

Faculty recognized the need and expectation to teach students and residents as a part of their role in an academic or “teaching hospital” but described the challenges of balancing teaching with other obligations. Faculty expressed confusion regarding where teaching should fit into competing demands, sometimes viewing teaching as a community obligation (“pulling weight”), an act of service (“generous”), or even an indulgence (“guilty pleasure”).

Teaching requires extra effort at times, and that can sometimes impact how you schedule your clinic and subsequently your clinical productivity. (P5)

It seems like it's so fun to get to teach, but there's always a trade-off, either that you don't have enough time then for the patients or for this work or that work…Everybody in the hospital setting feels this tension. (P8)

[Before NTEACH] I was thinking about [teaching] more like something I was like trying to fit in that I wanted to do more of, but that I didn't necessarily have space or permission to do. (P11)

Faculty feared the potential of competing demands to erode engagement in the educational mission, given “so many other things going on” and “lots of asks on your time” and described weighing the amount of time required for various teaching endeavors when choosing whether to engage in teaching.

#### Made Teaching Visible — “Not Just a One-Off”

Faculty noted that teaching is typically hidden work, not highlighted to colleagues, the department, or even oneself. By making faculty teaching activities visible, NTEACH added value to the teaching endeavor. Faculty appreciated seeing the entirety of the educational effort and seeing the effort reinforced as a part of one's professional roles and identity.

Seeing that in the numbers and being like, “Wow, that's what I've done,” …It's a source of validation that I wouldn't otherwise have…It feels like an exposure of the efforts that I do. (P2)

It's been a great experience for me that it's continued to show me that I like to do it… that this is an activity that I enjoy as part of my profession. (P6)

I think with NTEACH, it made the teaching seem more legitimate, especially because it's all these little bits and pieces. It's not like I had the time or bandwidth or opportunity to be like a core instructor…It made it seem more like, “Okay, this is a real thing that you're doing. Not just one off… scraps here and there.” (P11)

#### Directed Faculty Toward Eligible Teaching

Faculty reported spending less than 10 minutes per month logging their teaching and appreciated monthly reminders linking to the mobile platform. Faculty, particularly new faculty who were less familiar with the breadth of educational programs, used NTEACH to learn about which types of teaching were most valued by the department. Some noted the potential to improve faculty recruitment for teaching that reduced RVU productivity, such as outpatient clinic teaching, whereas others raised concern about the potential to devalue ineligible teaching.

I saw the list of the different things, and I realized there were some that I didn't know about. And so I reached out and said, “I would be happy to help with this or that thing”… [It] has just encouraged me to do more because I felt the nod from work that it was okay for me to do [it]. (P11)

It also shows some of the breadth of where the department needs additional help with teaching…about certain things that I want to do, if that could potentially be a part of what the department needs. (P7)

#### Communicated the Department Values Education

Faculty described the impact of the monetary incentive itself, including its potential to relieve financial stress, offset lost clinical productivity, or justify additional time spent during the evenings or weekends on work perceived as overflow from teaching roles. Some faculty used the incentive to pay for professional development or to support wellness activities.

…when I'm teaching…I see fewer patients…I think it's nice to have some type of compensation for that. (P9)

I think it just reinforces it and makes it seem like something that the department treasures. And treasures, maybe a strong word, something that the department understands it needs. And yeah, I think it almost, it legitimizes the position to say we don't take for granted what we expect of people and that we actually want to show that being an educator is something that we value, and we value it financially too… (P7)

To feel that gesture of appreciation and recognize like, “Hey, the department values what I'm doing in a real way, and here it is.” (P3)

#### Reinforced Identity, Pride, and Belonging 

Faculty were consistent that the NTEACH program supported their educator identity, pride, and sense of belonging in a manner that transcended the direct monetary impact by communicating that their educational work was valued. Faculty acknowledged that by communicating departmental values, NTEACH enabled faculty educators to experience alignment of personal and departmental priorities. Although participating faculty often noted that their identities as educators were well-established before enrollment, the program reinforced their educator identity, their sense of belonging within the department, and their pride in their department. Faculty perceived that NTEACH was unique among academic neurology programs, reflecting leadership esteem for education and educators, suggesting a viable pathway for faculty advancement, and encouraging faculty educator roles.

…it validates that what I've selected as my career choice is one that is aligned with what our department values. (P10)

I think it reinforces and supports my identity as a teacher, like a medical educator, because like I said, a lot of it is volunteer…And I think that NTEACH, encourages you to do more teaching because it's incentivized…For me, I think it's a reinforcement. (P9)

I'm proud to work in a department that values education in this way. (P8)

Faculty cited untapped opportunities to expand nonmonetary support for educators through supporting advancement, well-being, and development, including the potential for leadership to create ways of giving back time (e.g., email-free or meeting-free half days). The ability to foster excellence in education was acknowledged as a challenge for incentive programs, and faculty cited the unintended consequence of undervaluing teaching and leadership roles that were not included in the program, like writing letters of recommendation, fellowship and faculty teaching, on-the-fly teaching, and mentoring. Faculty noted that careful consideration should be given to equity when including incentivized teaching for which some faculty may not be eligible to participate. And in general, faculty expressed a desire for the expansion of community-building activities such as education symposia, faculty and staff awards and spotlights, opportunities to share best practices, and informal or social gatherings. At the time of this article, we continue to expand community events and the budget and scope of NTEACH, which has just completed its largest year-to-date.

## Discussion

As faculty face increasing pressures to generate revenue in our complex and evolving health care system, academic neurology programs should consider mechanisms to support and sustain their education mission and educators. Since mission-based management of education was first proposed by the AAMC in the 1990s, many EVU systems have been described and summarized.^2^ Program goals have generally sought to align incentives with priorities, with a variable emphasis on other factors such as increasing faculty engagement, scholarship, satisfaction, and performance. Uptake has been slowed by barriers, including challenges with data collection, lack of alignment with quality metrics, and administrative burden. More recently, there have been increasing concerns regarding the complex impact of extrinsic rewards on faculty motivation.^19,21^ Among motivational theories, self-determination theory posits that individuals are intrinsically motivated to act (e.g., educators teaching out of personal desire) and that extrinsic factors (e.g., incentive payments) could undermine intrinsic motivation by implying that personal desire is insufficient or not present.^18^ Careful thought needs to be given to the alignment of teaching incentives with the desired behavior to ensure rewards are meaningful and avoid devaluing the behavior or other activities unintentionally, and incentives should avoid becoming impersonal or replacing efforts to support through other means such as by fostering a community of practice for faculty educators. We described our process for developing a feasible and sustainable teaching incentive program at an academic neurology center and for evaluating the impact on faculty engagement, motivation, and identity.

We addressed several key challenges in the development of our program that others can learn from while acknowledging that there is no single right program and that each will necessarily be unique to local needs (Table 4). (1) Those seeking to develop an EVU program should engage in a change management process that includes the creation of a guiding coalition and programmatic vision with broad stakeholder engagement. The vision should include a process to measure, build on, and communicate early wins. (2) Because teaching activities are myriad, the challenge of defining program scope requires clear program goals that align with the educational mission and a teaching inventory to define eligible teaching activities. (3) In addition, because the teaching endeavor is evolving, there should be a process for clear and transparent iterative review and transparent communication with faculty. Clear communication minimizes the potential for unintended or false equivalence in a system that generates “relative value” for teaching efforts. (4) Feasibility for faculty engagement and data management are key considerations and common barriers. We found that faculty will engage in programs that rely on self-reporting and that there may be additional benefits of this approach, shifting funding to faculty who find it most valuable while minimizing administrative burden. (5) For programs like ours that focus on funding direct teaching using a time metric, the question of additional metrics and outcomes may be intentionally integrated into the program, such as the promotion of quality and support for advancement. We included faculty development time in our program as a mechanism to support excellence, but other metrics of excellence could include having a minimum teacher evaluation rating or expectations for timely learner evaluations, among others. We added faculty appreciation letters to support advancement. (6) Programs should have a plan for sustainability, which we accomplished through a committee consisting of faculty educators, finance, and departmental leadership (NCOST). Developing a cadre of faculty committed to the program and willing to serve on NCOST has been essential given the inevitable evolution of our educational programs and priorities. Finally, (7) program evaluation should include measures of faculty experience, such as impact on motivation, identity as educators and engagement, to ensure that programs are meeting the needs of those who teach and the needs of institutions for teachers.

**Table 4:** Key Considerations When Building a Teaching Incentive Program

Consideration	Key points
Apply a change management framework	Apply a change management process including a guiding coalition with stakeholder representation, programmatic vision, and process to measure and build on early wins
Define programmatic goals and eligibility	Define goals for your program that align with the departmental education priorities, create a teaching inventory, define eligible teaching and teachers
Engage in iterative review	Create and share the process for iterative review with stakeholders; consider equity and relative value for various activities
Ensure feasibility for faculty and program	Develop a mechanism for faculty engagement that reduces barriers to enrollment and enables data collection with minimal administrative burden
Consider quality and advancement	Integrate mechanisms that can address quality metrics into the program (faculty development or teaching evaluation data) and support faculty advancement (thank you letters for promotion packets)
Plan for sustainability	Create a plan for sustainability of the program. Engage faculty, leadership, finance, and technology in the iterative review process
Evaluate effect on faculty	Given programmatic goals to support teachers, evaluation plan should include impact on faculty factors (such as motivation, identity, and engagement)

Our faculty reported several factors that affected their engagement, motivation, and identity and help to conceptualize the impact of NTEACH on faculty (Figure). They described engaging in an ongoing calculus regarding their teaching in the face of competing demands on their time. Teaching incentive programs, like NTEACH, have the potential to reinforce the educator identity by making hidden teaching visible so that it becomes more than the sum of its parts. In addition, opt-in programs can be designed to be relatively time-efficient and allow for faculty who find the program valuable to self-select to enroll, potentially directing funding to faculty in need of support. The incentive payment itself reduces stress and promotes wellness, and teachers experience the funding as a meaningful reflection that their department values education. Education incentive programs, therefore, serve not only to direct faculty to engage in the core educational mission of the department but also help faculty to feel valued as educators and to experience a sense of belonging and pride that reinforces their educator identity. Faculty cautioned about the potential to devalue ineligible teaching and highlighted unmet needs for additional community building among clinician educators.

**Figure:**
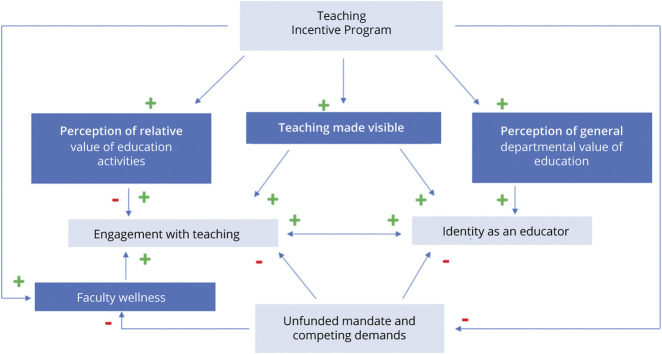
NTEACH Impact on Faculty EVU = education value unit; NTEACH = Neurology Teaching EVU and Community Hub.

This work has several limitations. First, we describe a program within a single department at a single institution. The experience at our institution may not apply to other institutions depending on the alignment of mission, program design, support from leadership, or other factors. In addition, although faculty may choose to adjust clinical targets prospectively based on this program because our program did not directly reduce clinical productivity expectations, we cannot comment on the impact of that design feature on clinical revenue or faculty experience. Second, our program evaluation focuses on faculty perceptions regarding the impact on their identity and motivation to teach. We cannot, therefore, comment on the impact of the program on other outcomes such as teaching quality or ease of faculty recruitment. In addition, given 55% of faculty completed the survey, although this is a typical faculty response rate, we cannot exclude the possibility of selection bias affecting our program evaluation. The same concern could be raised regarding the 11 completed interviews, although our approach to thematic analysis and saturation of themes minimizes this threat. Finally, our program focused on a more limited scope of essential, direct teaching that did not enrich clinical productivity and did not include support for scholarship or leadership roles. Our experience may not be analogous to those developing programs with a different focus. Given that approximately 25% of our faculty participated and enrollment was optional, we may be supporting the subset of faculty that are most interested in teaching and feel they would benefit from support to do so. This could be seen as a weakness or a strength of the design.

For those seeking to develop teaching incentive programs at academic neurology centers, we acknowledge the complexities of program design and describe a program and principles for successful implementation that allow for feasibility and sustainability. In addition, we find that although careful thought should be given to the complex potential impact of external rewards on intrinsic motivation, there remains a place for monetary incentive programs to effectively support faculty motivation and identity as educators.

## Glossary

AAMC: Association of American Medical Colleges
EVU: education value unit
NCOST: Neurology Committee on Sustainable Teaching
NTEACH: Neurology Teaching EVU and Community Hub
RVU: relative value unit
